# Ontogeny of small RNA in the regulation of mammalian brain development

**DOI:** 10.1186/1471-2164-15-777

**Published:** 2014-09-09

**Authors:** Sharon L Hollins, Belinda J Goldie, Adam P Carroll, Elizabeth A Mason, Frederick R Walker, Darryl W Eyles, Murray J Cairns

**Affiliations:** School of Biomedical Sciences and Pharmacy, Faculty of Health and Medicine, the University of Newcastle, University Drive, Callaghan, NSW 2308 Australia; Centre for Translational Neuroscience and Mental Health, Hunter Medical Research Institute, Newcastle, NSW 2305 Australia; Queensland Brain Institute, University of Queensland, Brisbane, Qld 4072 Australia; Queensland Centre for Mental Health Research, Wacol, Qld, 4076 Australia; Schizophrenia Research Institute, Sydney, NSW Australia

**Keywords:** Cortex, Interactions, MicroRNA, mRNA, Neurogenesis

## Abstract

**Background:**

MicroRNAs (miRNAs) play a pivotal role in coordinating messenger RNA (mRNA) transcription and stability in almost all known biological processes, including the development of the central nervous system. Despite our broad understanding of their involvement, we still have a very sparse understanding of specifically how miRNA contribute to the strict regional and temporal regulation of brain development. Accordingly, in the current study we have examined the contribution of miRNA in the developing rat telencephalon and mesencephalon from just after neural tube closure till birth using a genome-wide microarray strategy.

**Results:**

We identified temporally distinct expression patterns in both the telencephalon and mesencephalon for both miRNAs and their target genes. We demonstrate direct miRNA targeting of several genes involved with the migration, differentiation and maturation of neurons.

**Conclusions:**

Our findings suggest that miRNA have significant implications for the development of neural structure and support important mechanisms that if disrupted, may contribute to or drive neurodevelopmental disorders.

**Electronic supplementary material:**

The online version of this article (doi:10.1186/1471-2164-15-777) contains supplementary material, which is available to authorized users.

## Background

The developing cerebral cortex is highly compartmentalized and involves precise regulation of cell proliferation, migration and differentiation [1, 2]. The behaviour of cells undergoing these neurodevelopmental processes is governed by spatiotemporal changes in gene expression [3]. Translation and intracellular traffic of these transcripts is influenced by small non-coding miRNA that function as molecular guide sequences for ribonucleoprotein complexes involved in targeted mRNA degradation or translational repression (reviewed [4–7]). By regulating mRNAs post-transcriptional fate, miRNA subsequently play a significant role in organizing complex patterns of gene activity. Thus these small RNA molecules also display distinct expression profiles [8–10], and exhibit both temporal and spatial specificity [8, 11–13]. For example, *miR-9* has been demonstrated to play a role in controlling neurogenesis timing via targeting of progenitor-promoting and cell-cycle exit-promoting genes [14]. Accordingly, its expression has been shown to increase during neurodevelopment, but not in the postnatal period [11], and to be regionally restricted to telencephalic, diencephalic and tectal periventricular proliferative zones [12].

Analysis of miRNA and their target mRNA expression in neuronal cell cultures supports a regulatory role for miRNAs in neuronal development [15–18]. Consistent with this role, a number of neural-specific or neural-enriched miRNAs have been identified [19] and are thought to be involved in regulating early brain development (reviewed [20]). For example, *miR-134* and *miR-137* regulate neuronal maturation through the modulation of their target mRNA. The brain-specific *miR-134* regulates dendritic spine development by repressing the translation of an mRNA encoding a protein kinase, *Limk1*, that controls spine development [21], while the brain-enriched *miR-137* has a significant role in neuronal maturation and dendritic morphogenesis via direct targeting of *Mib1*, a ubiquitin ligase important for neurogenesis and neurodevelopment [22]. This supports existing evidence for the role of miRNAs in neurogenesis [23], morphogenesis [24], neuronal cell specification [25] and oligodendrocyte differentiation and myelination [26].

In the current study we wanted to determine if miRNA and their predicted targets are altered in accordance with their developmental stage and neurodevelopmental regionalization. More specifically we compare developmental miRNA and gene expression in the caudal regions including the mesencephalon and metencephalon that develop relatively early, with more rostral areas such as the telencephalon and diencephalon which develop later [27], as we hypothesize that miRNA are the subject of related spatiotemporal regulation. By matching miRNAs with their inversely-regulated mRNA targets, we suggest that miRNA’s post-transcriptional regulatory influence changes throughout neurodevelopment, with a pattern of expression in the developing telencephalon occurring after that of the mesencephalon in accordance with their respective cytoarchitectural and synaptic maturity.

It is critical for the development and use of rodents as animal models of neuropsychiatric disorders that we have a good understanding of the nature and timing of regional differentiation in the rodent brain. Predicting neurodevelopmental events and recognizing differing windows of vulnerability to environmental insults will help develop clinically relevant experimental models.

## Results

### Temporal dynamics of miRNA expression in the telencephalon and mesencephalon between E12 and P0

**Figure 1:**
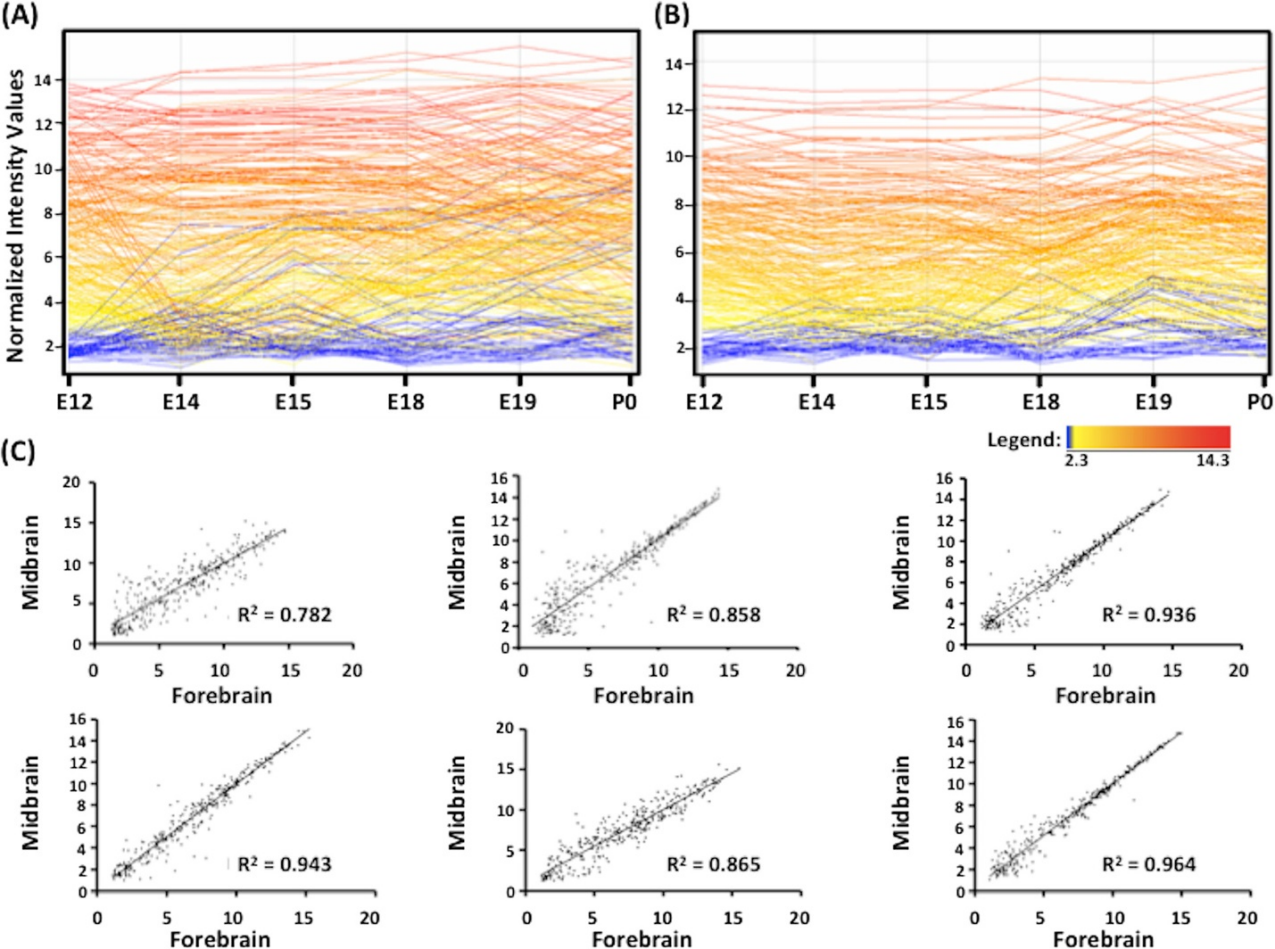
**miRNA expression profiles in the developing rat brain.** Intensities of differentially expressed miRNA microarray probes across development in the telencephalon **(A)** and mesencephalon **(B)**. Each colored line represents one probe. Normalized signal intensities range from 2.3 to 14.3 as indicated by color legend. Color legend is relative to E12 such that probes with low expression at E12 (signal intensity of 2.3) are in blue while those with high expression at E12 (signal intensity of 14.3) are in red. **(C)** Scatter plots illustrating miRNA expression in the mesencephalon and telencephalon at each timepoint. Solid line = line of best fit; R^2^ = Pearson correlation coefficient.

**Figure 2:**
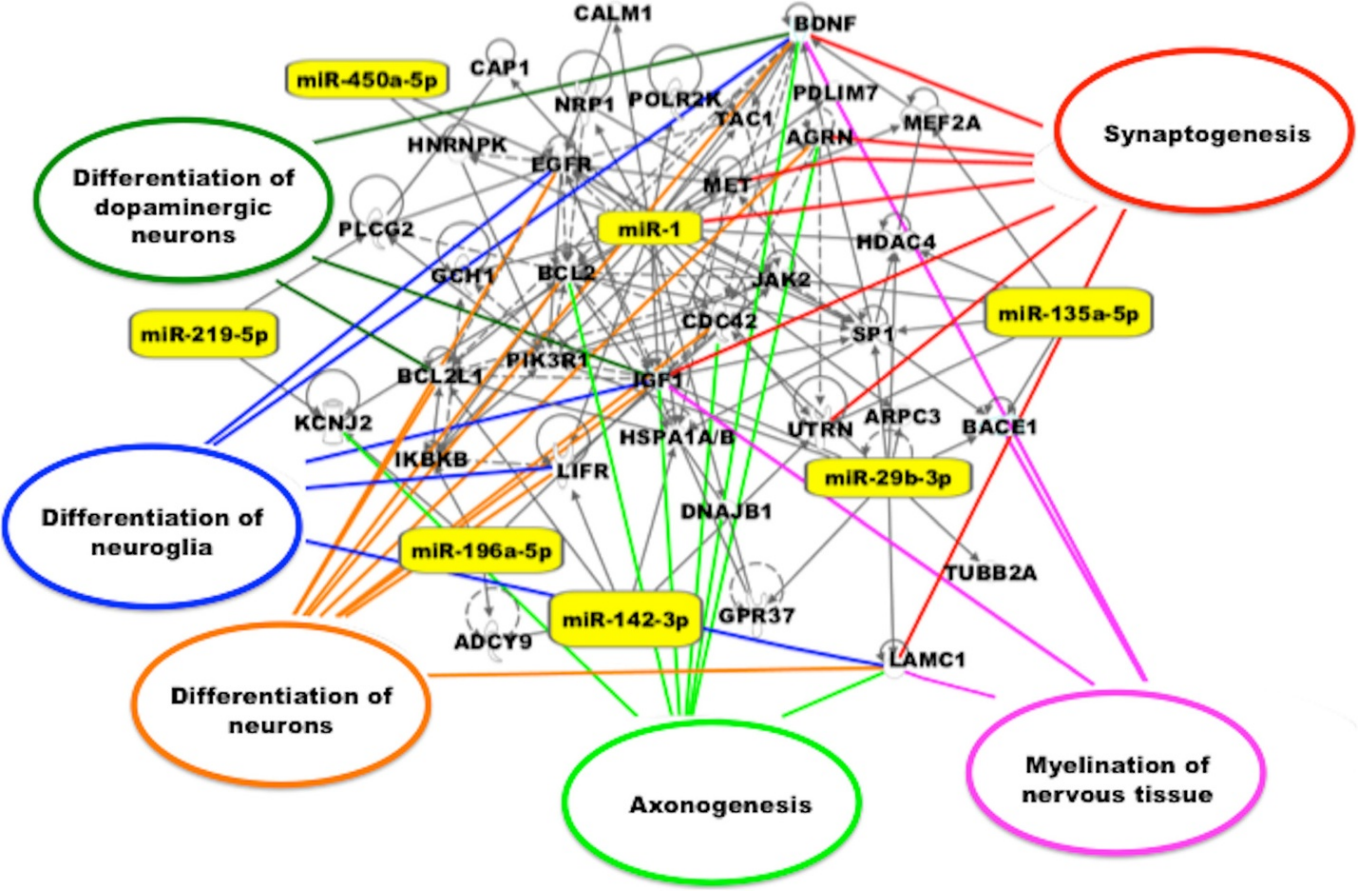
**Computational gene interaction predictions: network of E12 telencephalon-specific miRNA involved in the Neurotransmitters and other nervous system signalling pathways.** A network of putative gene targets of the 32 telencephalon-specific miRNA at E12 was constructed with the Ingenuity Systems Pathway Analysis (IPA) software. Solid grey lines specify direct relationships whereas dotted grey lines indicate indirect interactions. Genes involved in select neurodevelopmental processes are indicated with solid color lines.

miRNA expression was analysed in the telencephalon and mesencephalon of embryonic (E) (E12, E14, E15, E18, E19) and newborn (postnatal (P) day 0) rats using GeneChip miRNA array matrices (Affymetrix). Data analysis revealed 303 miRNA in the telencephalon and 266 in the mesencephalon with differential expression during brain development. miRNA expression displayed greater variation in the telencephalon (Figure 1A) compared to the mesencephalon (Figure 1B) throughout development (Figure 1C), with 97 miRNA in the telencephalon having substantive temporal variance (SD > 1.3) across development as compared to 38 miRNA in the mesencephalon. There were also a number of miRNA with altered expression specific to the telencephalon. This occurred at E12 (32 miRNA) and E19 (26 miRNA) only. In contrast the mesencephalon had no region-specific miRNA expression at any time during the developmental timepoints examined. Furthermore we also observed stage-associated expression in the telencephalon-specific miRNA, with 13 and 7 expressed only at E12 or E19 respectively (Additional file 1). To gain an appreciation of the biological implications of these telencephalon-specific miRNA during early telencephalon development, putative target genes of the 32 miRNA specific to the telencephalon at E12 and the 26 miRNA specific to the telencephalon at E19 were assessed for Kyoto Encyclopedia of Genes and Genomes (KEGG) pathway enrichment using the Gene Annotation Tool to Help Explain Relationships (GATHER) online database. For the telencephalon-specific miRNA at E12 this revealed a number of significantly enriched pathways with relevance to neural connectivity and synaptic plasticity, such as focal adhesion; adherens junction; oxidative phosphorylation; regulation of actin cytoskeleton and ECM-receptor signalling. Similarly, the telencephalon-specific miRNA at E19 were predicted to be enriched in the Mitogen-activated protein (MAP) kinase signalling pathway and Wingless/int (Wnt) signalling pathway (Additional file 2). Further analysis using Ingenuity Pathway Analysis (IPA) software (Ingenuity Systems) revealed roles in important neurodevelopmental processes including: the differentiation of dopamine neurons; neuron and neuroglia differentiation; myelination; synaptogenesis; and axonogenesis (Figure 2).

**Figure 3:**
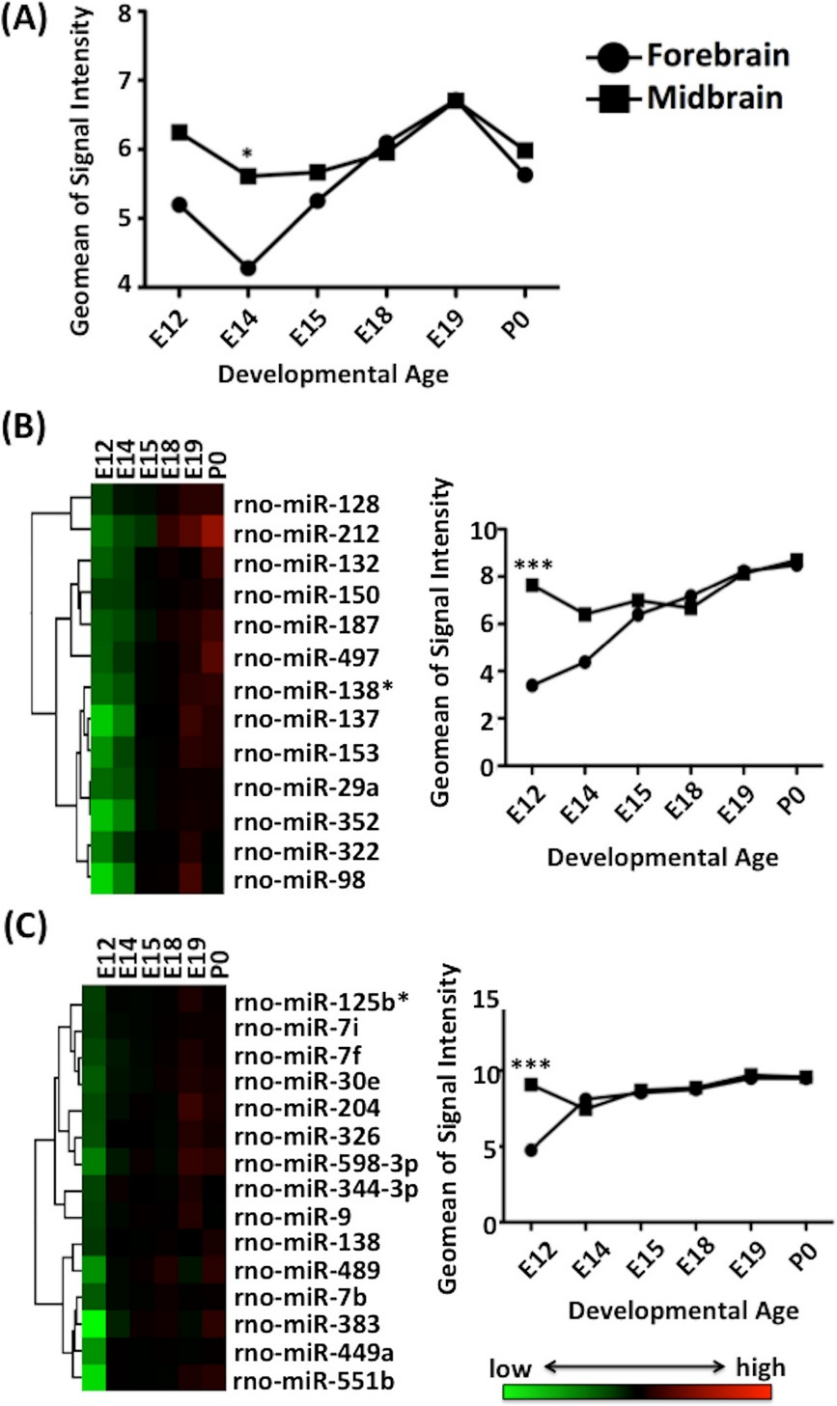
**miRNA expression in the developing rat brain. (A)** Expression of the 97 miRNA showing strong developmental regulation in the telencephalon and mesencephalon throughout development (for display purposes expression was derived by calculating the geomean of signal intensity values for all dynamic miRNAs at each developmental timepoint). All data were analysed for significance by one-way ANOVA, followed by Tukey-Kramer’s *post hoc* t-testing (* = p < 0.05). **(B & C)** Relative expression levels for the 97 miRNA showing strong development regulation were hierarchically clustered (supervised, correlation uncentred, average linkage; Cluster 3.0). Colors indicate relative signal intensities. (Java Treeview; http://jtreeview.sourceforge.net). Two major clusters are shown in six columns corresponding to the six time points. Comparison of expression of telencephalon and mesencephalon miRNA for each cluster is shown adjacent to the heatmap. All data were analysed for significance by one-way ANOVA, followed by Tukey-Kramer’s *post hoc t*-test. *** = p < 0.001.

To determine those miRNA that were differentially regulated with respect to developmental age, all miRNA were ranked based on their temporal expression variance through E12 to P0. miRNA undergoing substantial changes throughout development (SD > 1.3) were selected for downstream analysis (Additional file 3). The 97 miRNA identified were found to have significantly lower expression (F = 5.271, n = 12, p < 0.05) in the telencephalon compared to the mesencephalon at embryonic day 14 (Figure 3A). Supervised hierarchical clustering of these miRNA was characterized by distinct expression clusters, two of which showed significantly lower expression (p < 0.001) in the telencephalon compared to the mesencephalon at embryonic day 12 (Figure 3B & C). Prominent among this group were miRNAs that are enriched in neurons and associated with developmental regulation (*let-7i, let-7f, let-7b, miR-98*), cell cycle regulation (*miR-137, mIR-128*) and neural activity (*miR-132, miR-212*). Bioinformatic analysis of these 97 miRNA through IPA (Ingenuity Systems) identified 9652 putative miRNA target genes. Gene ontology (GO) function and KEGG pathway enrichments were performed by mapping the predicted target genes from the developmentally regulated miRNAs using the GATHER online database. For these miRNAs and their predicted targets, 22 GO functions and 3 KEGG pathways were significant (Bayes factor >6). The top ontologies included: regulation of cell cycle; development; morphogenesis; regulation of transcription; and neurogenesis (Additional file 4).

In summary, while we observed substantial overlap between miRNA expressed in the telencephalon and mesencephalon, there was a number of functionally significant miRNA expressed exclusively in the telencephalon. From longitudinal analysis in each brain region, it was also apparent that telencephalon miRNA expression was subject to more temporal variation compared to the mesencephalon. Genes predicted to respond to this developmental variation in telencephalon cortical miRNA expression were enriched in pathways associated with neural development and function.

### Developmental regulation of mRNA expression in the telencephalon and mesencephalon between E12 and P0

mRNA expression was analysed in the telencephalon and mesencephalon of embryonic (E12, E14, E15, E18, E19) and newborn (P0) rats using RatRef-12 Expression BeadChips (Illumina). Data analysis revealed a total of 16,691 genes with differential expression during neural development (E12-P0), comprised of 14,371 genes in the telencephalon and 13,906 genes in the mesencephalon. To determine those genes that were differentially regulated with respect to developmental age, all mRNA were ranked based on their temporal expression variance between E12 to P0. Those that demonstrated the greatest variation throughout development (SD >3.2) were selected for bioinformatic evaluation (Additional file 5). These 100 genes with high temporal expression dynamics were further assessed for their involvement in biological pathways. GO function and KEGG pathway enrichment of these genes revealed 43 significant (p < 0.05) GO functions and 1 KEGG pathway (Additional file 6). Network analysis using IPA (Ingenuity Systems) for predicting potential interactions between the differentially expressed genes generated three networks with the highest IPA analysis scores (47, 25 and 21) including Cell-To-Cell Signalling and Interaction; Nervous System Development and Function; Cellular Growth and Proliferation; and Cellular Development (Table 1). While these biological processes accorded well with those predicted by the developmentally regulated miRNA, we wanted to explore the possibility that these 100 dynamically expressed genes were most likely responding to changes in miRNA expression and driving developmentally significant changes in cell morphology and physiology, by bioinformatically associating the putative interactions with observed changes in predicted relationships or miRNA-mRNA interactions.

**Table 1:** **Network analysis using IPA**

Associated Network Functions	Score
Cell-To-Cell Signalling and Interaction, Nervous System Development and Function, Cell Death	47
Cellular Growth and Proliferation, Haematological System Development and Function, Haematopoiesis	25
Cellular Development, Cell Signalling, Organ Development	21
Cell Morphology, Cellular Function and Maintenance, Cell Cycle	19
Psychological Disorders, Cell Death, Gene Expression	13

Network analysis using IPA (Ingenuity Systems) for predicting potential interactions between the differentially expressed genes potentially regulated by differentially expressed miRNA.

### miRNA-mRNA interactions

Given that miRNAs predominantly function to negatively regulate gene expression, we examined the biological significance of inverse expression patterns between differentially expressed miRNA and differentially expressed genes in the telencephalon during development. We used Ingenuity’s miRNA Target Filter® to analyze all possible miRNA and target-gene interactions from the miRNA and gene expression array data sets. The results were filtered based on the confidence of interaction and an inverse miRNA to target mRNA relationship. These gene sets were then functionally annotated using the Database for Annotation, Visualization, and Integrated Discovery bioinformatics resources 6.7 (DAVID) online database, revealing significant over-representation in neural pathways throughout development. This included axon guidance, neuroactive ligand-receptor interaction, MAP kinase signalling, Wnt signalling, long-term depression and calcium signalling (Additional file 7).

**Figure 4:**
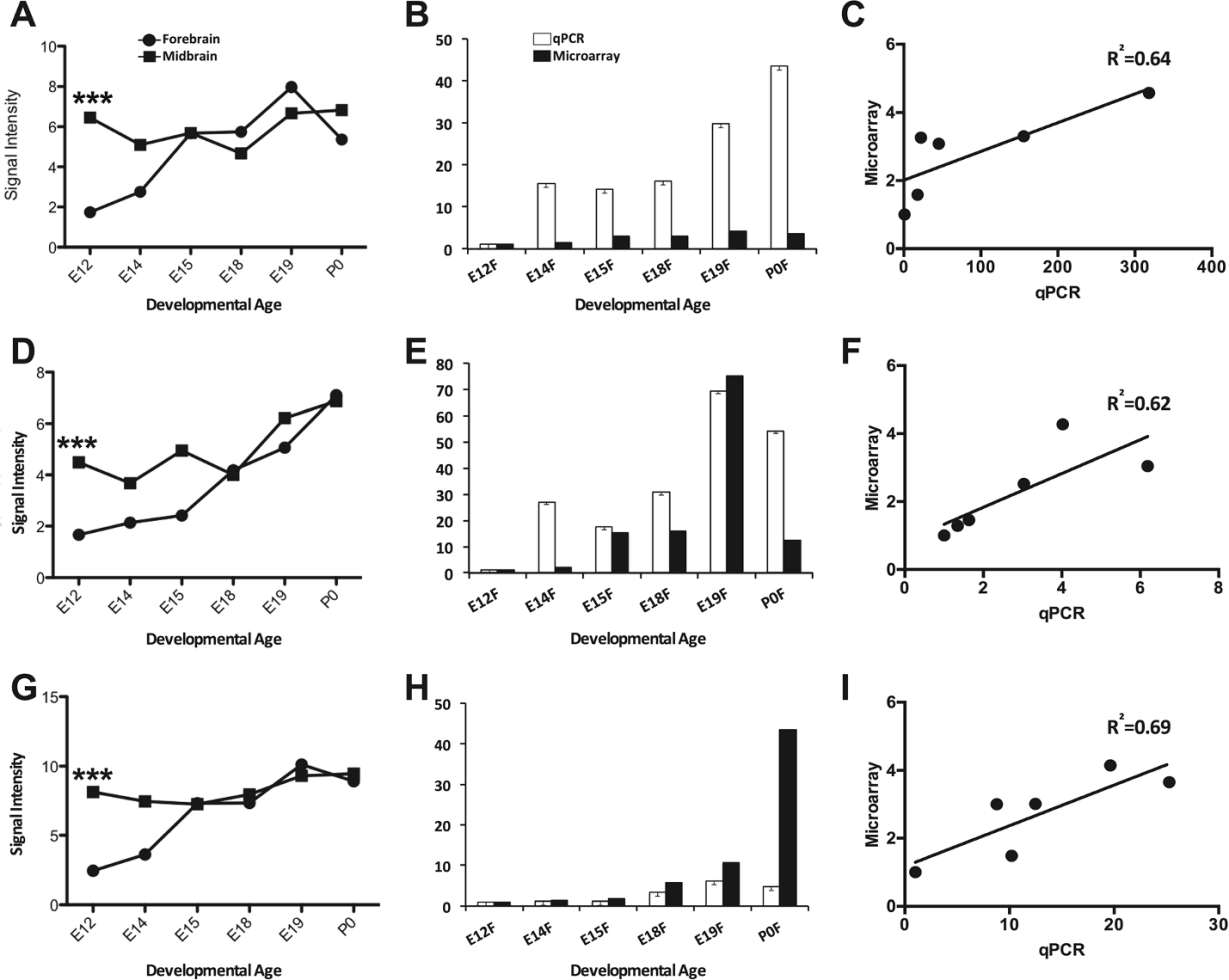
**Select miRNA expression in the developing rat brain.** Total RNA was isolated from rat telencephalon and mesencephalon at E12, E14, E15, E18, E19 and P0, amplified, labelled and hybridised to Affymetrix Genechip miRNA 1.0 microarrays. Data were normalised using cubic spline and analysed using GeneSpring GX11. Figures show the normalised signal intensity values for *miR-98* **(A)** and *miR-212* **(D)** and *miR-137* **(G)** at each developmental stage in the mesencephalon and telencephalon (*** = p < 0.001). Findings were validated by Q-PCR and data expressed as a fold-change. Mean value of E12 was set to 1-fold induction, and mean values of all other ages were related to E12. MiRNA expression as determined by microarray appears as black bars and Q-PCR results appear as white bars. For Q-PCR, relative miRNA expression in the telencephalon was determined by the difference between their individual cycle threshold (Ct) value and that produced in the same sample for the U6 snRNA (∆Ct). Error bars represent standard deviation of the means. Microarray expression data was validated by RT-PCR for *miR-98* **(B)**, *miR-212* **(E)** and *miR-137* **(H)** with correlation coefficients of 0.64 **(C)**, 0.62 **(F)** and 0.69 **(I)** respectively.

We further investigated the putative roles of *mir-98, mir-212* and *mir-137* in the development of the telencephalon. All three miRNA were determined to be dynamically regulated with more than a three-fold increase in telencephalon expression between E12 and E19. These neuronally important miRNAs have critical roles in neurodevelopment and are associated with neuropathology (see Discussion). Concordantly, their predicted gene targets were also highly significant in our study. Validation of microarray expression for these three miRNA using quantitative real-time reverse transcription PCR (Q-PCR) (Figure 4) confirmed significantly lower expression in the telencephalon compared to the mesencephalon at E12. To further investigate their biological relevance, IPA software was used to identify putative differentially expressed target genes of these dynamically regulated miRNA. Analysis of this gene dataset using the DAVID online database revealed significant (p < 0.05) over-representation of neurodevelopmental functions in the mammalian forebrain cortex, including axonogenesis; neuron projection development; synaptic transmission; forebrain development; regionalization; and learning and behaviour (Additional file 8).

### Target gene silencing *in vitro*

**Figure 5:**
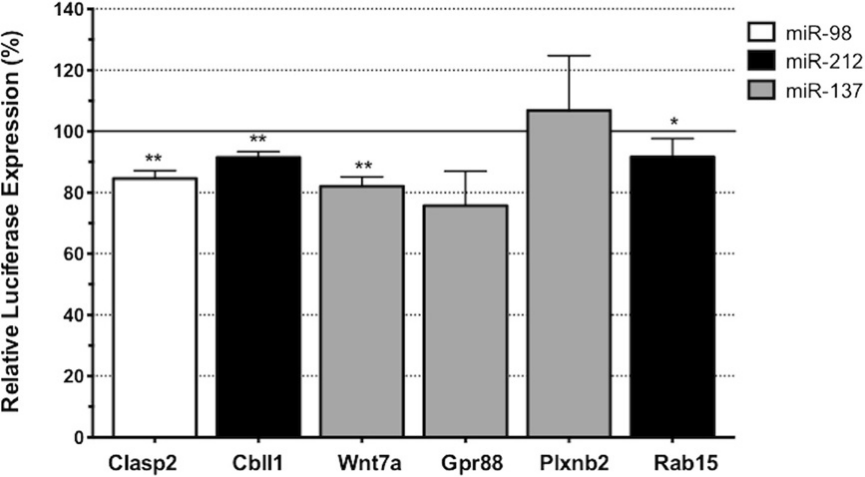
**Regulation of neuronal development-associated reporter gene constructs by miRNA.** Chart shows relative activity of reporter gene constructs (x-axis) in response to co-transfected miRNAs. Relative luciferase activity for each reporter/miRNA combination was expressed as a percentage of the response to scrambled controls. Bars are mean ± SEM. (** = p < 0.01, * = p < 0.05).

Our expression analysis of miRNA and their cognate mRNA targets suggested that there are a tremendous number of interactions that underlie the spatiotemporal segmentation in mammalian brain development. However, to further support the functional association between neural development-associated target genes and altered expression in this group of miRNAs, the respective miRNA recognition elements (MRE) from a selection of six functionally significant target genes, *Clasp2, Rab15, Cbll1, Wnt7a, Gpr88* and *Plxnb2*, were cloned into the 3’ untranslated region (UTR) of a luciferase reporter gene construct and co-transfected into a recipient cell line with synthetic miRNA mimics corresponding to those altered *in vivo*. The degree of reporter gene activity and the impact of miRNAs were then ascertained by measuring the relative luciferase activity (Figure 5). MiRNAs interact with their respective MREs in the 3’UTR of transcripts and each gene may contain binding sites for multiple miRNA. Therefore observing miRNA-mediated effects using a reporter gene construct allows subtle changes in gene expression to be observed. Most of the reporter constructs behaved in accordance with expectation and were repressed 8-25% (p < 0.05) in the presence of synthetic miRNA in comparison to a scrambled-duplex co-transfected control. These data indicate that transfected miRNAs bound to their target MREs and repressed the expression of luciferase, with the most responsive targets derived from the 3’-UTR of *Wnt7a* and *Gpr88*. These were both targeted by *miR-137*, which appeared to have the greatest overall effect, whereas *miR-212* had the least effect on these target gene constructs. The only exception was the *Plxnb2* construct, which displayed increased expression in the presence of synthetic *miR-137*. Collectively, these reporter assays supported the existence of relationships between genes reported to be associated with neural development and developmentally regulated miRNAs.

## Discussion

### miRNA and post-transcriptional regulation of neurodevelopment

The trajectory of human brain development closely mirrors that seen in the rodent [28] and as such the rat offers an excellent model to investigate the coordination and timing of miRNA expression across fetal development. In the current study we examined the expression profiles of miRNA within the mesencephalon and telencephalon from E12 until birth. We hypothesized that the developing telencephalon would exhibit a significantly different temporal miRNA profile in relation to the mesencephalon and this was supported by the observation that 87% of expressed miRNA underwent significant change across development, with clear differences in patterning between the two regions. The highest level of change in miRNA expression occurred early in development (E12-E15) and plateaued thereafter, with differences between the telencephalon and mesencephalon decreasing noticeably during that time period. In accordance with our hypothesis, telencephalon miRNA expression was significantly lower than the mesencephalon at E12 and consistent with the delayed development of this region. We also observed 32 miRNA with predicted function in neurodevelopmental processes, exclusively expressed in the telencephalon during early brain development (E12).

We then employed a bioinformatics approach to further understand the differential developmental trajectories of these two brain regions. Temporally regulated miRNAs and genes were examined using a combination of miRNA and gene expression profiling and computational target prediction. Hierarchical clustering of miRNA during telencephalon development revealed two distinct clusters of miRNA. These clusters had significantly higher expression in the mesencephalon at E12 whilst expression in the telencephalon remained significantly lower (Figure 3B & C). The miRNA contained in these clusters are predicted to target temporally-regulated genes involved in brain signalling pathways important for neurodevelopment and neuronal functioning including: dopamine receptor signalling; GABA receptor signalling; glutamate receptor signalling and neuroactive ligand-receptor interactions. As expression of the neuroactive ligand-receptor interactions pathway is demonstrated to correlate with synaptic maturity [29], this data is consistent both with the telencephalon being less differentiated at this time point and more importantly implicates a critical role for miRNA in this process. This also indicates that at such an early timepoint, the developing telencephalon may be largely unaffected by environmental insults compared to the mesencephalon. This may be particularly relevant for certain developmental animal models of disease. For instance the widely used maternal immune activation model of schizophrenia employs the artificial viral construct poly I:C which induces a strictly time-limited inflammatory response of approximately two days in the maternal animal [30]. This may be why when it is commonly administered at E9 in mice (equivalent to E10 in a rat), the downstream effects may be restricted to the developing mesencephalon rather than later maturing regions such as the telencephalon [31].

### miRNA-mRNA interactions

While there are some exceptions, miRNA function is usually associated with repression of target gene expression, such that when a miRNA is expressed at high levels, its target mRNA is reduced [32–34]. Indeed, several reports have identified inverse expression patterns between differentially expressed miRNA and differentially expressed mRNA during development [17, 18]. In this study we found the putative down-regulated gene targets of up-regulated miRNA in the telencephalon between E12 and E14 are predicted to be involved in many important brain signalling pathways including axon guidance, neuroactive ligand-receptor interaction, calcium signalling and long-term depression.

Altered expression of *miRs-98*, -*212* and -*137* was particularly noteworthy, as these three neuronally important miRNAs were much more abundant in developing mesencephalon than telencephalon at the earliest embryonic age showing more than a three-fold increase in telencephalon expression between E12 and E19. All three miRNA have critical roles in neurodevelopment and are associated with neurological disorders such as Alzheimer’s disease and schizophrenia. We confirmed a functional link between these three miRNA and suppression of expressed target genes by cloning the MREs from *Clasp2, Rab15, Cbll1, Wnt7a, Gpr88* and *Plxnb2* downstream of a luciferase reporter gene. These reporter constructs were then co-transfected with synthetic analogues of their cognate miRNA into a recipient cell line, such that the extent of reporter gene expression and therefore the influence of miRNA could be ascertained through the measurement of relative luciferase activity (Figure 5). Most of these constructs were significantly repressed in the presence of synthetic miRNA. In particular, regulation of 3’-UTR elements from *Wnt7a* (*miR-137*) and *Gpr88* (*miR-137*) was particularly strong and, along with *Clasp2* (*miR-98*) and *Rab15* (*miR-212*) provides support for the role of post-transcriptional regulation in axon and dendritic growth, maturation and function [35–38]. Pathway analysis of these target genes indicated that these miRNA may exert significant influence during neurodevelopment through the regulation of pathways including: axon guidance; neuroactive ligand-receptor interaction; Wnt; Erythroblastic Leukemia Viral Oncogene Homolog (ErbB); and MAP kinase signalling.

In the telencephalon, expression levels of *miR-98* increased over four-fold between E12 and E19, a similar finding to that of Sempere and colleagues [15], who found *mir-98* expression levels progressively accumulated upon neuronal differentiation in both mouse and human embryonic carcinoma cells. Experiments by Thomson and colleagues [39] similarly found levels of *miR-98* were significantly increased in mice at E14.5 compared to levels in embryonic stem cells. *Mir-98* is a member of the let-7 family, highly conserved across species in sequence and function and involved in the developmental timing of cell fates (reviewed [40]). In support of a role for *miR-98* in telencephalon development, *mir-98* overexpression was found to significantly decrease the activity of a luciferase reporter gene fused to the cytoplasmic linker associated protein 2 (*Clasp2*) 3’-UTR. *Clasp2* plays a crucial role in chromosome segregation during mitosis [41] and is a key regulator of neuronal morphogenesis, neuronal polarity and synapse formation and activity [37].

Similarly, the results suggest a role for *miR-212* in brain development with expression levels increased more than four-fold in the telencephalon between E12 and birth. *MiR-212* arises from the miR-212/132 cluster, which is highly conserved in vertebrates. *MiR-212* and *miR-132* are tandem miRNAs with similar target specificity due to their identical seed sequences. In the brain, *miR-212* and *miR-132* play vital roles in the formation and plasticity of neuronal connections, and long-term synapse activation (reviewed [42, 43]). Dysregulation of *miR-212* is associated with drug addiction [44], neuropathological disorders [43] and schizophrenia [45]. Our luciferase reporter assays support *miR-212* involvement in neurodevelopment via regulation of Rab15, member RAS oncogene family (*Rab15*) and Cbl proto-oncogene-like 1, E3 ubiquitin protein ligase (*Cbll1*). *Rab15* belongs to the Rab family of low molecular weight (LMW) GTP-binding proteins, crucial in ensuring the spatiotemporal regulation of membrane traffic [46]. *Rab15* is enriched in neural tissue and plays a role in regulating neurotransmitter release [47]. *Cbll1* is a RING finger type E3 ubiquitin ligase and an important regulator of cell proliferation [48]. In Drosophila, *Cbll1* is required for proper function of cell surface proteins and for endoderm and mesoderm morphogenesis [49]. Consistent with the increase of *miR-212* expression between E12 and E19, *Cbll1* function is demonstrated to be most vital in the early stages of embryogenesis, with its contribution declining in later stages [49].

Another developmentally regulated miRNA is *miR-137*, with expression levels increasing more than four-fold in the telencephalon between E12 and E19. *Mir-137* is enriched in neurons and involved in neuronal maturation, regulation of dendritic development and phenotypic maturation of new neurons [22]. Recent studies demonstrate increasing expression of *miR-137* during neuronal differentiation, leading to reduced neural stem cell proliferation, accelerated neural differentiation and decreased dendritic development [22, 50]. Dysregulation of *miR-137* has been associated with intellectual disability and Alzheimer’s Disease [51, 52] and the *MIR137* gene has also recently found to be highly associated with schizophrenia in a genome wide screen and associated with cognitive function in patients with the disorder [53, 54]. This study confirmed the potency of *miR-137* to down-regulate G-protein coupled receptor 88 (*Gpr88*) and wingless-type MMTV integration site family, member 7A (*Wnt7a*) using a reporter gene strategy. *Gpr88* is a brain-enriched G protein–coupled receptor with a role in modulating the striatal dopaminergic system [55] and in regulating medium spiny neuron excitability [38]. *Wnt7a* signalling is critical to regulate dendritic spine growth and synaptic strength [35], mediating synapse density and numbers, and hippocampal network structure [56]. During development Wnt signalling is required for cell proliferation and differentiation, cell polarity generation and embryonic patterning.

As various brain regions develop at different times there is likely to be differing windows of vulnerability to environmental insults. Appropriate spatiotemporal expression of miRNA is vital during brain development and dysregulation during sensitive stages is likely to have a profound impact on the coordinated expression of developmentally significant genes associated with the pathophysiology of neuropsychiatric disorders. This is supported by the alterations of miRNA expression observed in the neuropathology of Alzheimer’s disease [51], Huntington’s disease [57, 58], Parkinson’s disease [59–61] and schizophrenia [62, 63]. Understanding the role of miRNA in brain ontogeny could identify critical windows of developmental significance that are susceptible to environmental insults which contribute to the development of neurobehavioural or neuropsychiatric syndromes.

## Conclusions

This study represents the first global characterization of both miRNA and mRNA expression between the telencephalon and mesencephalon at various stages during development in rodent brain. Comparing the telencephalon and mesencephalon affords a molecular basis for understanding the nature and timing of regional differentiation. Although these results may have been hypothesised based on current evidence suggesting that the mesencephalon develops earlier than the telencephalon, the present data now provide direct molecular support that differential regional and temporal miRNA expression within the brain is part of this process. Knowledge of the temporal and spatial expression profiles of miRNA and their target genes is an important initial step in elucidating their functions. Together these results show that miRNA expression is frequently region- and time-specific with an important influence in functionally relevant pathways in the developing brain.

## Methods

### RNA preparation and analysis of integrity

Telencephalon and mesencephalon were dissected from brains of embryonic (E) (E12, E14, E15, E18, E19) and newborn (P0) rats. Each time point represents a pooled litter of rat embryos (n = 8-12). Animals were handled in accordance with the University of Queensland's animal care and ethics committee approval, in compliance with the current Australian Law. Total RNA was isolated from the telencephalon and mesencephalon using TRIzol reagent as described previously [64]. RNA concentration, integrity and purity were assessed using the Agilent 2100 Bioanalyser (Agilent Technologies). RNA Integrity Numbers (RIN) were automatically calculated with the provided system software [65]. All samples showed RIN values superior to 8.5.

### MicroRNA expression profiling

For miRNA profiling, total RNA (700-1000 ng) was labelled using a FlashTag Biotin HSR RNA labelling kit according to manufacturer’s instructions (Genisphere). Labelled RNA was hybridised to the Affymetrix Genechip miRNA 1.0 microarrays (miRBase version 11.0) for 16 hours at 48°C and 60 rpm in a hybridisation oven (Affymetrix) and washed and stained with the Fluidics Station 450 (Affymetrix). Hybridisation, wash and stain reagents were from the Hyb, Wash and Stain kit (Affymetrix). Arrays were scanned with Affymetrix GeneChip Scanner 3000 7G. Differential miRNA expression was analysed using GeneSpring GX 11.0 (Agilent Technologies) and was assessed as having a minimum 2-fold difference in either direction between two time-points. To determine those miRNA that were differentially regulated with respect to developmental age, the standard deviation of each miRNA across development (E12-P0) was calculated. MiRNA with a standard deviation of >1.3 were considered to be developmentally regulated.

### mRNA expression profiling

Total RNA was prepared for microarray using the RNeasy Mini Kit (Qiagen) and amplified with the Illumina TotalPrep RNA Amplification Kit (Ambion). Microarrays were performed using the Illumina Rat-Ref-12_V1 Expression BeadChip, following the manufacturer's instructions, and scanned using the Illumina BeadArray Reader and BeadScan software (Ambion, Scoresby, VIC, Australia). Bead summary data were normalized in GenomeStudio v3 (Illumina) using Cubic Spline. Differential mRNA expression was analysed using GeneSpring GX 11.0 (Agilent Technologies) and was assessed as having a minimum 2-fold difference in either direction between any two time-points.

### Target gene and pathway analyses

miRNAs that were determined to be differentially expressed were further analysed for putative target genes under their influence using Ingenuity® microRNA target filter [Ingenuity® Systems (http://www.ingenuity.com)]. Pathways and gene ontology enrichment analysis of the target genes was performed using the DAVID (http://david.abcc.ncifcrf.gov/) [66, 67] and GATHER (http://gather.genome.duke.edu/) [68] online databases. To further investigate biologically relevant targeting, differential miRNA and mRNA expression data was analyzed through the use of Ingenuity® microRNA target filter to generate lists of interactions between genes and miRNAs. Results were filtered based on a high confidence of interaction and an inverse miRNA to target mRNA expression relationship. The identified genes were then subjected to pathways and gene ontology enrichment analysis.

### Quantitative real-time reverse transcription PCR

Validation of differentially expressed miRNA was performed by Q-PCR on a subset of the differentially expressed miRNA. Briefly, 500 ng of sample RNA was treated with DNase-I (Invitrogen), and multiplex reverse transcription was performed with Superscript II reverse transcriptase (Invitrogen), a 3 nmol/L mix of miRNA sequence specific primers and primers for U6 small nuclear RNA. The primer sequences for the amplification of specific primers were as follows: rno-miR-98 - 5′-GTAAAACGACGGCCAGTAACAATA-3′ (forward), 5′-TGAGGTAGTAAGTTG-3′ (reverse); rno-miR-212 - 5′-TAA + CAGTCTCCAG-3′ (forward), 5′-GTAAAACGACGGCCAGTCTACGCG-3′ (reverse); rno-miR-137 - 5′-TT + ATT + GCTTAAGAATA-3′ (forward), 5′-GTAAAACGACGGCCAGTCTACGCG-3′ (forward); U6 snRNA 5′-TGCTTCGGCAGCACATATAC-3′ (forward), 5′-AGGGGCCATGCTAATCTTCT-3′ (reverse). Triplicate reactions were set up in a 96-well format with the epMotion 5070 automated pipetting system (Eppendorf, Hamburg, Germany) and carried out with the Applied Biosystems (Foster City, California) 7500 real-time PCR machine. Relative quantification was assessed using the formula 2^-Δ^CT. The delta Ct was calculated by subtracting the Ct of the endogenous control (geometric mean of U6 small nuclear RNA (snRNA)) from the Ct of the miRNA.

### Reporter construction

To generate reporter vectors bearing binding sites for the three miRNA examined in detail, *miR-98, miR-212* and *miR-137*, oligonucleotides encoding target gene miRNA recognition elements (MREs) were annealed to form *SpeI* and *HindIII* restricted overhangs of a ligatable cassette compatible with *SpeI* and *HindIII* digested pMIR-REPORT vector (Ambion, Austin, TX) as described previously [69, 70]. Recombinant reporter constructs were amplified in chemicompetent DH5alpha Escherichia coli bacteria cells (Bioline, Sydney) and purified using endotoxin free minipreps (Promega, Madison, WI) before being verified by DNA sequencing (AGRF, St. Lucia, QLD, Australia) and quantitated using a NanoDrop 2000 (Thermo Scientific, Delaware, ME, USA).

### Cell culture

Human embryonic kidney (HEK)-293 cell cultures were maintained as confluent monolayers at 37°C with 5% CO_2_ and 90% humidity in Dulbecco’s modified Eagles medium (DMEM) with 10% (vol/vol^-1^) fetal calf serum, 20 mM HEPES (4-(2-hydroxyethyl)-1-piperazineethanesulfonic acid), 0.15% (wt/vol^-1^) sodium bicarbonate and 2 mM L-glutamine.

### Transfection

For the luciferase assay, HEK-293 cells were seeded into a 96-well plate at a density of 20,000 cells per well and transfected 24 h later using Lipofectamine 2000 (Invitrogen). In each case transfections were performed according to the manufacturer’s instructions, with 100nM synthetic miRNA (Invitrogen). Validation of predicted target genes was accomplished by co-transfecting HEK293 cells with synthetic miRNA and recombinant firefly luciferase reporter gene constructs containing 3’-UTR sequences substituted from the target gene. Each reaction was performed in quadruplicate and replicated at least once. To validate the putative MREs, the HEK293 cell line, which is of embryonic origin and easily transfected, was an ideal candidate for the investigation of the functional regulation of miRNA. Candidate genes were selected based on: their inverse expression respective to their targeting miRNA; their role in neurodevelopment and association with neuropathology; a high MRE-binding specificity score using the miRanda online software [71] (*Clasp2* – 142; *Rab15* – 158; *Cbll1* – 161; *Wnt7a* – 149; *Gpr88* – 150; *Plxnb2* – 161).

### Reporter gene assay

Twenty-four hours after transfection, cells were harvested in 100 ml Passive Lysis Buffer (Promega, Madison, WI) and 10 ml lysate was used to measure the relative luciferase activity using the dual-luciferase reporter assay kit (Promega, Madison, WI). Reporter gene silencing in response to miRNA co-transfection was monitored with respect to a pRL-TK control plasmid expressing renilla luciferase using a Synergy plate reader with dual reagent injector (Biotek). To control for the nonspecific effects associated with transfection, the controls were co-transfected with mutant miRNAs or mutant anti-miRs (Invitrogen).

### Statistical analysis

Statistical significance analysis was assessed using one-way or two-way analysis of variance (ANOVA) with Benjamini Hochberg multiple comparisons testing (Prism software version 5.0; GraphPad Software Inc., San Diego, CA). A value of p < 0.05 was considered significant. The Tukey-HSD test with a level of significance set at p < 0.05 was used for post hoc comparisons. Unsupervised hierarchical clustering was performed in Cluster (Stanford University, Palo Alto, CA, USA) [72]. Data were log transformed and median centred by genes. Genes and arrays were clustered, correlation uncentred, by average linkage clustering and visualized through Java Treeview V.1.1.6r2 (http://jtreeview.sourceforge.net) [73]. Statistical comparison of luciferase data sets was performed by two-tailed paired Student’s *t*-test.

## Electronic supplementary material

Additional file 1: **A table listing miRNA expressed in the telencephalon but not the mesencephalon at E12 and at E19.** Also listed are miRNA that are specific to the telencephalon at E12 only and at E19 only. (XLSX 50 KB)

Additional file 2: **A table listing putative pathways regulated by the E12 and E19 telencephalon-specific miRNA.** Enriched pathways of putative target genes of the 32 miRNA specific to the telencephalon at E12 and the 26 miRNA specific to the telencephalon at E19 were identified using the GATHER online database. Putative gene targets were identified using IPA. Only those pathways and ontologies with a Bayes factor >6 were considered significant. (XLSX 42 KB)

Additional file 3: **A table listing the 97 dynamically regulated miRNA.** Also listed are the standard deviation values for each miRNA in both the forebrain and midbrain. (XLSX 49 KB)

Additional file 4: **A table listing enriched ontologies of putative target genes of the 97 dynamically regulated miRNA identified using the GATHER online database.** Putative gene targets were identified using IPA. Only those pathways and ontologies with a Bayes factor >6 were considered significant. (XLSX 9 KB)

Additional file 5: **A table listing the 100 dynamically regulated mRNA.** Also listed are the standard deviation values for each mRNA in both the forebrain and midbrain. (XLSX 46 KB)

Additional file 6: **A table listing enriched ontologies of the 100 dynamically regulated miRNA identified using the GATHER online database.** Only those pathways and ontologies with a Bayes factor >6 were considered significant. (XLSX 10 KB)

Additional file 7: **A table listing enriched pathways of differentially expressed miRNA targeting differentially expressed RNA identified using the DAVID online database.** Only those pathways with a p-value <0.05 were considered significant. (XLSX 14 KB)

Additional file 8: **A table listing predicted ontologies enriched with target genes of** ***miRs-98, -212*** **andX -** ***137.*** Enriched ontologies of putative target genes of *miR-98, miR-212* and *miR-137* were identified using the DAVID online database. Putative gene targets were identified using an intersection of the miRanda, IPA and TargetScan output. n, number of input genes in pathway.%, percentage of total target genes analysed that are represented in this pathway. (XLSX 11 KB)

## Abbreviations

miRNA: microRNA
mRNA: Messenger RNA
E: Embryonic
P: Postnatal
KEGG: Kyoto Encyclopedia of Genes and Genomes
GATHER: Gene Annotation Tool to Help Explain Relationships
MAP: Mitogen-activated protein
Wnt: Wingless/int
IPA: Ingenuity pathway analysis
GO: Gene ontology
DAVID: Database for annotation, visualization, and integrated discovery
Q-PCR: Quantitative real-time reverse transcription PCR
MRE: miRNA recognition elements
UTR: Untranslated region
RIN: RNA Integrity Numbers
HEK: Human embryonic kidney
SD: Standard deviation.
